# 73. The role of rituximab in the treatment immunoglobulin G4-related disease: a case report

**DOI:** 10.1093/rap/rky034.036

**Published:** 2018-09-20

**Authors:** Fathelrahman Osman, Josephine Vila

**Affiliations:** 1 Infectious Disease/Microbiology, Royal Victoria Infirmary/Freeman Hospital, Newcastle upon Tyne, UNITED KINGDOM; 2 Rheumatology Department, Freeman Hospital, Newcastle upon Tyne, UNITED KINGDOM

**Introduction:** Immunoglobulin G4-related disease (IgG4-RD) represents an immune-mediated fibro-inflammatory condition resulting in mass forming lesions with characteristic histopathological appearances. The disease can affect multiple different organs, with a range of clinical presentations to different medical and surgical specialties.

**Case description:** We describe a case of a 71-year old female who presented with refractory facial pain following a dental procedure. She was treated with repeated courses of antibiotics by the dentist and was subsequently referred for an ENT opinion. A CT scan revealed complete opacification of the left maxillary sinus with bony erosion of the floor, medial, anterior and posterior aspects of the maxillary antrum. Histological biopsies were reported as showing eosinophilic angiocentric fibrosis with an IgG4:IgG ratio of > 40%, consistent with a diagnosis of IgG4-RD. Peripheral blood IgG4 levels were normal and extended autoantibody screening, including ANCA, viral and TB screening were negative. Past medical history was significant for an unexplained episode of bronchus intermedius stenosis, ten years earlier. Thoracotomy yielded multiple biopsies which were reported at the time as showing non-specific granulomatous inflammation. Analysis of the historic samples revealed elevated levels of IgG4 staining. The patient was referred to rheumatology and commenced prednisolone 30mg orally with some improvement in facial pain. The dose was subsequently weaned to a maintenance dose of 7.5mg daily. Mycophenolate mofetil (MMF) was commenced as a steroid sparing agent (1 gram twice daily). Unfortunately, there was radiological evidence of disease progression with ongoing bone loss, despite this therapy and the decision was made in the IgG4-RD MDT, to proceed with Rituximab with cyclophosphamide induction. There has been some resolution of the inflammatory changes and treatment is ongoing.

**Discussion:** With an estimated incidence of 60m per million populations, IgG4-RD is a rare, but increasingly recognized condition. First line therapy remains glucocorticoids. Rituximab has been commissioned by NHS England for use in refractory or organ threatening IgG4-RD. In this case rituximab has shown superiority to MMF in improving the symptoms and resolution of inflammation. Arguably the trial of MMF delayed the more effective treatment and resulted in more bony loss. The extent to which the addition of iv cyclophosphamide potentiated the effects Rituximab is not known and more research is needed. The episode of bronchus intermedius stenosis suffered almost a decade earlier, coupled with the multiple courses of antibiotics and delayed contemporary diagnosis, highlights the diagnostic challenges of IgG4-RD.

**Key Learning Points:** Consider further review and IgG4 staining of any available previous histopathology samples if a diagnosis of IgG4-RD is made. Consider the addition of cyclophosphamide in organ threatening disease as an induction agent. Consider Rituximab early in organ threatening disease to prevent progression to irreversible damage.

**Disclosure: F. Osman:** None. **J. Vila:** None.

